# Pharmacological blockade of the mast cell MRGPRX2 receptor supports investigation of its relevance in skin disorders

**DOI:** 10.3389/fimmu.2024.1433982

**Published:** 2024-10-18

**Authors:** Colin H. Macphee, Xinzhong Dong, Qi Peng, Daniel V. Paone, Per Stahl Skov, Katrine Baumann, Theresa Roethke, Deborah A. Goldspink, Samuel K. Pearson, Zining Wu

**Affiliations:** ^1^ Research and Development, GSK, Collegeville, PA, United States; ^2^ Solomon H. Snyder Department of Neuroscience, Johns Hopkins University School of Medicine, Baltimore, MD, United States; ^3^ Howard Hughes Medical Institute, Johns Hopkins University School of Medicine, Baltimore, MD, United States; ^4^ RefLab ApS, Hvidovre, Denmark; ^5^ GSK, Medicines Research Centre, Stevenage, United Kingdom

**Keywords:** mast cell, MRGPRX2, skin, neuropeptides, substance P, antagonist

## Abstract

**Introduction:**

Because MRGPRX2 is now recognized as the mast cell receptor for basic secretagogues, there is currently a tremendous interest in whether MRGRPX2 could play an important role in various pruritic dermatoses such as chronic spontaneous urticaria. Therefore, we sought to identify new potent and selective antagonists to pharmacologically characterize the biological role of MRGPRX2.

**Methods:**

Various relevant *in vitro*, *ex vivo*, and *in vivo* model systems were used to investigate the role of MRGPRX2. This included the study of freshly isolated human skin mast cells and human basophils as well as an *ex vivo* human skin microdialysis preparation. The additivity of MRGPRX2 and FcεR1-mediated degranulation was also investigated. Human MRGPRX2 knock-in mice were generated to interrogate pharmacokinetic/pharmacodynamic relationships because both antagonists studied were shown to be human specific.

**Results:**

Two novel and structurally distinct MRGPRX2 antagonists were identified with one, Compound B, being orally active and demonstrating high potency in blocking Substance P–mediated degranulation using freshly isolated human skin mast cells with half maximal inhibitory concentration (IC_50_) at 0.42 nM. Compound B also potently blocked Substance P–stimulated histamine release from resident mast cells in a human skin explant setup as well as blocking itch in an established behavioral scratching model using MRGPRX2 knock-in mice. Unlike human mast cells, Substance P failed to elicit a functional response in human basophils.

**Conclusion:**

These data fully support the investigation of MRGPRX2 receptor antagonists in mast cell–driven allergic skin disorders such as chronic spontaneous urticaria.

## Introduction

Mast cells are immune cells of the hematopoietic lineage thought to have multifaceted functions during homeostasis and in various disease states. Mast cells evolutionarily predate the mammalian adaptive immune system and are noted for their longevity compared with most immune cells. Mature granulated mast cells are most numerous in the tissues that not only line inner and outer body surfaces but also populate most other tissues in lower density. Notably, mast cells are found lining blood vessels and are found associated with nerve fibers (1, 2).

Mast cells express many different receptors to allow them to sense and react to a wide diversity of stimuli. Classically, they are activated by Immunoglobulin E (IgE) antibodies binding to the Fc epsilon receptor 1 (FcεRI) receptor, leading to secretion of a wide range of mediators with both local and systemic effects, including histamine, serotonin, proteases, prostaglandins, chemokines, and cytokines (1, 3). Significant attention over recent years has focused on the mast cell–specific receptor for basic secretagogues, MRGPRX2. Following decades of searching for the responsible receptor, this changed when the G protein–coupled receptor (GPCR) MRGPRB2, the ortholog of human MRGPRX2, was identified as the sole mast cell receptor for basic secretagogues in mice (4).

MRGPRX2 is now recognized as a multiligand receptor able to respond to a variety of exogenous and endogenous cationic substances, collectively called basic secretagogues, including the established exogenous mast cell basic secretagogue, Compound 48/80 (Cpd 48/80) (5, 6). The most potent ligands for MRGPRX2 are dominated by endogenous neuropeptides, including Substance P (SP) and adenylate cyclase–activating polypeptide (PACAP). Less potent agonists include endogenous antimicrobial peptides as well as secreted eosinophil products that critically share cationic properties needed for receptor recognition. In addition, MRGPRX2 is recognized and activated by a variety of pharmacological agents, such as icatibant and atracurium, causing pseudo-allergic reactions (4).

Various published observations using knockout mice are consistent with the Mrgprb2/MRGPRX2 receptor playing a role in mast cell–mediated neurogenic inflammation. For instance, Mrgprb2/MRGPRX2 agonists induce various symptoms such as flushing, swelling, and itch in wild-type mice but not in Mrgprb2-deficient mice (4, 7). Mrgprb2-deficent mice have also demonstrated significant reductions in inflammation (leukocyte infiltration, including mast cells), swelling, pain, itch, and overall clinical score in various disease models (8, 9). As such, MRGPRX2 is attracting a great deal of attention as a putative drug target for the treatment of a variety of mast cell–driven pathologies, including disorders of the skin (5, 10, 11).

Therefore, we sought to identify potent and selective antagonists to pharmacologically characterize the role of MRGPRX2 in relevant *in vitro*, *ex vivo*, and *in vivo* models to support further investigation of the role of this mast cell receptor in skin disorders. Although MRGPRX2 has been considered a mast cell–specific receptor (12), basophils have been reported to constitutively express functionally active MRGPRX2 (13). Thus, we also investigated whether basophils from both allergic and non-allergic individuals could respond to MRGPRX2 activation.

## Materials and methods

### Materials

Cortistatin 14, SP, and PACAP (1–27) were purchased from GenScript. PAMP-12 was purchased from R&D Systems. C3a was purchased from Complement Technology. The following materials were used: pNAG (p-nitrophenyl N-acetyl-β-D-glucosamide, Sigma-Aldrich), methyl Cellulose (Sigma, M0262-100G), Cpd 48/80 (Sigma, C2313-250MG), Phosphate-buffered Saline (PBS) (Thermo Fisher, 10010023), Fibronectin human plasma (Sigma, F0895-2MG), HEPES 1 M buffer (Corning, Life Sciences, MT 25-060-CI), Hank's Balanced Salt Solution (HBSS) (Thermo Fisher, 14065056), Percoll (Sigma, P4937-500ML), Dulbecco’s modified Eagle medium (DMEM; Thermo Fisher, 11995073), fetal bovine serum (FBS; Sigma, F4135), murine stem cell factor (SCF; PeproTech, 250-03), Fluo-4 AM (Thermo Fisher, F14201), SP (stock, 10 mM in dimethyl sulfoxide (DMSO); Tocris), and goat polyclonal anti-human IgE (ϵ-chain specific) antibody (stock, 1 mg/mL in PBS; Sigma, I6284). Compounds were dissolved in DMSO at 1 mM or 10 mM.

### *In vitro* cell systems

Cell culture reagents were purchased from Thermo Fisher Scientific unless otherwise stated. The LAD2 human mast cell line was in-licensed from National Institutes of Health (NIH) and routinely cultured in StemPro™-34 SFM with nutrient supplement, 1× GlutaMax™, and recombinant human SCF (100 ng/mL). HEK293-MRGPRX2/Gα15 cells were cultured in DMEM containing 10% FBS. The parental U2OS cells were culture in DMEM/F-12 containing 10% FBS. BacMam virus containing human MRGPRX2 gene or animal orthologs [mouse Mrgprb2 (Gene ID: 243979), rat Mrgprb3 (Gene ID: 691194), dog MRGPRX2 (Gene ID: 102125889), cynomolgus monkey MRGPRX2 (Gene ID: 692078), and rhesus monkey MRGPRX2] were used to transduce U2OS cells for transient expression.

A Ca^2+^ mobilization assay was used to screen for small-molecule compounds with agonist or antagonist activity against human MRGPRX2 using HEK293-MRGPRX2/Gα15 cells. Two million small-molecule compounds in GSK collection were screened at single concentration of 10 μM on FLIPR^TETRA^ (Molecular Devices) instruments in dual detection mode for both activation and inhibition activities. Cortistatin 14 at approximately EC_80_ concentration was used as the activation ligand for the inhibition phase of the high-throughput screening. Compounds of interest were followed up and characterized in concentration response curves to determine their mode of activity, potency, and selectivity.

Mouse peritoneal mast cells were purified and imaged as previously described (4). Mast cells were resuspended at 5 × 10^5^ to 1 × 10^6^ cells/mL in DMEM with 10% FBS and recombinant mouse SCF (25 ng/mL) and plated onto glass cover slips coated with fibronectin (30 µg/mL). For imaging, after 2 h of incubation at 37°C, 5% CO_2_, mast cells were loaded with Fluo-4 along with 0.02% Pluronic F-127 for 30 min at room temperature, washed three times in calcium imaging buffer (CIB), and used immediately for imaging. Cells were used within 2 h of loading. Cells were identified as responding if the [Ca^2+^] rose by at least 50% for >10 s.

### Isolation of human skin mast cells

The human biological samples were sourced ethically, and their research use was in accord with the terms of the informed consents under an Institutional Review Board/Ethics Committee (IRB/EC) approved protocol.

Tissue was processed, removing adipose and cutting into small segments. Epidermis was removed after incubation with dispase II (2 mg/mL; Sigma-Aldrich) [100 mL/50-cm^2^ tissue, base buffer; Dulbecco’s Phosphate-buffered Saline (D-PBS) (Gibco, supplemented with Ca^2+^/Mg^2+^), amphotericin B (2.5 μg/mL), penicillin (100 units/mL)/streptomycin (100 μg/mL) (Sigma-Aldrich)] for 37°C for 2–4 h followed by 4°C overnight. The remaining dermis was homogenized into a uniform mass and digested with an enzyme cocktail [collagenase IV (10 mg/mL; Gibco), hyaluronidase I-S (5 mg/mL; Sigma), DNAse I (10 μg/ml; Roche)] in base buffer (supplemented with 10% FBS) in a shaking incubator at 37°C for 90 min. Digested supernatant was passed through a 100-μm cell strainer to remove undigested tissue, and cells were pelleted by centrifugation (400 g, 10 min). Cells were then resuspended in base buffer (supplemented with 10% FBS) and left on ice. Supernatant was added back to the undigested tissue and incubated for a further 90 min at 37°C. This process was repeated and both digests were combined and resuspended in D-PBS (Gibco) supplemented with 10% FBS and 2 mM EDTA and passed through a 70-μm cell strainer to achieve a single-cell suspension. Positive selection using a CD117 MicroBead Kit (Miltenyi) was then performed as per manufacturer’s protocol.

### LAD2 and human skin mast cell degranulation assays

Release of β-hexosaminidase from LAD2 cells was followed as a measurement of mast cell degranulation using a method described previously (4).

For human primary skin mast cell degranulation experiments, both β-hexosaminidase and tryptase were measured. Following isolation from primary human skin tissue, CD117^+^ cells were washed once in relevant assay buffer [β-hexosaminidase as above, and, for tryptase, HBSS (Sigma), both supplemented with 0.3% BSA] and then seeded into a conical bottom 96 well plate at 5 × 10^4^ cells per well. Cells were pre-treated with Compound B or a matched 0.2% DMSO (Sigma) vehicle in assay buffer and incubated at 37°C in 5% CO_2_ for 30 min. Cells were centrifuged (400g for 10 min), supernatant was removed, and appropriate treatments were added. Cells were incubated at 37°C in 5% CO_2_ for 1 h. Plates were centrifuged, with supernatants collected and lysates generated [cell pellets lysed in 0.1% Tx100 (Sigma), in assay buffer], and then stored at −80°C for later quantification. β-Hexosaminidase activity assay was performed as already described. For tryptase quantification: The amount of total tryptase (α and β tryptase) was quantified using the ImmunoCap Tryptase Test ran on either a Phadia 100 or 250 Immunoassay Analyzer as per the manufacturer’s protocol. Supernatants and lysates (reference wells were generated for each experiment) were diluted in sample diluent so that they could be interpolated from the standard curve.

### Screening, selection, and assaying of donor basophils

Blood bank buffy coats were screened for reactions against 10 inhalant allergens (grass, birch, mugwort, cat, dog, horse, *Dermatophagoides pteronyssinus*, *Dermatophagoides farinae*, *Cladosporium*, and *Alternaria*) and 10 food allergens (milk, egg, wheat, peanut, hazelnut, kiwi, cod fish, shrimp, celeriac, and soy). Five buffy coats with histamine release > 30 ng/mL to any of the tested allergens and with a histamine release to blood with anti-IgE > 30 ng/mL were included as allergic donors. Further, five buffy coats with histamine release < 10 ng/mL to any of the above-mentioned allergens and with a histamine release to blood with anti-IgE > 25 ng/mL were included as non-allergic donors.

The buffy coats were pre-incubated for 30 min at 37°C with either buffer or anti-human IgE in concentrations ranging from 0.04 µg/mL to 5 µg/mL. Subsequently, the samples were incubated 60 min at 37°C with SP in concentrations ranging from 0.2 µM to 25 µM. Following pre-incubation with anti-IgE and incubation with SP, released histamine was quantified as previously described (14). Briefly, histamine was bound to a glass fiber matrix at the bottom of microtiter plates and after a series of wash steps coupled to ortho-phtaldialdehyde before being detected fluorometrically in the Histareader™ 501 (RefLab ApS, Copenhagen, Denmark). Unspecific histamine release with baseline serum or buffer alone was <10 ng/mL corresponding to mean + 3 standard deviations of the histamine values measured without a specific stimulus (assay cutoff). Duplicate histamine determinations of each sample were performed. Assay variation was < 7%. Lower level of detection was 5 ng of histamine/mL. Histamine values are given as ng/mL histamine release after deduction of unspecific release.

### Human *ex vivo* skin microdialysis

Abdominal skin was sourced with informed consent from patients undergoing cosmetic surgery with ethical permission from the regional ethics committee (protocol number H-19088192). Microdialysis probes with a 3,000-kDa molecular weight cutoff (EP Medical, Copenhagen, Denmark) were primed for 1 h using a basal perfusate consisting of 0.1% human serum albumin in piperazine-N,N′-bis(2-ethanesulfonic acid) (PIPES) buffer to minimize non-specific adsorption to probe components.

Skin specimens measuring approximately 4 cm × 14 cm were excised from the parent skin sample, and subcutaneous fat was removed mechanically. The skin specimens were fixated on Styrofoam and guide cannulas (21G) were inserted intradermally at least 1 cm apart and ≥ 1 cm from the edge of the skin specimen. Ten probes were introduced into each skin specimen by means of the guide cannulas with an intradermal probe span of 20 mm. Probes were perfused at a flow rate of 3.0 μL/min (used throughout the experiment) for 30 min using basal perfusate in order to remove any unspecific histamine release caused by insertion trauma.

Next, the dialysate was collected for 10 min to assess basal skin histamine levels (“basal”). The probes were then switched to perfusion with a perfusate containing antagonists at different concentrations for 60 min to pre-treat the skin specimens and evaluate potential off-target effects, which could present as histamine release caused by antagonist itself (“pre-treatment”). After 1 h of pre-treatment, the skin was challenged using perfusion with a combination of antagonist and agonist (SP), and the dialysate was collected in 6 × 10-min intervals. Histamine in the dialysates was quantified as described above.

### MRGPRX2 knock-in mice and itch behavioral study

The generation and initial characterization of human MRGPRX2 knock-in (KI) mice has been previously reported (15). All experimental protocols were approved by the Animal Care and Use Committee at the Johns Hopkins University School of Medicine and studies conducted according to GSK’s Policy on the Care, Welfare and Treatment of Animals. All mice used for this study were 2- to 4-month old males and females (20–30 g) and randomized on body weight and sex using three groups of 18 animals per group.

The day prior to experimentation, human MRGPRX2 KI mice were habituated in the test chamber for 30 min undergoing a series of three mock injections. On the day of the experiment, animals were allowed to acclimatize to the test chamber for 10 min prior to injection. Mice received a single PO dose of Compound B (or vehicle) as a suspension in 1% methylcellulose at a dose level of 3 mg/kg (dose volume 10 mL/kg). Scratching behavior was monitored over 30 min following a single intra-dermal injection of 10 μL of Cpd 48/80 (10 μg/mL) or vehicle (saline) at 3 h post-dose of the antagonist. A bout of scratching was defined as a hindpaw directed continuous scratching movement at the injection site. The scratching behavior scorer was blinded to treatment.

### Statistical methods

**Figure 1:**
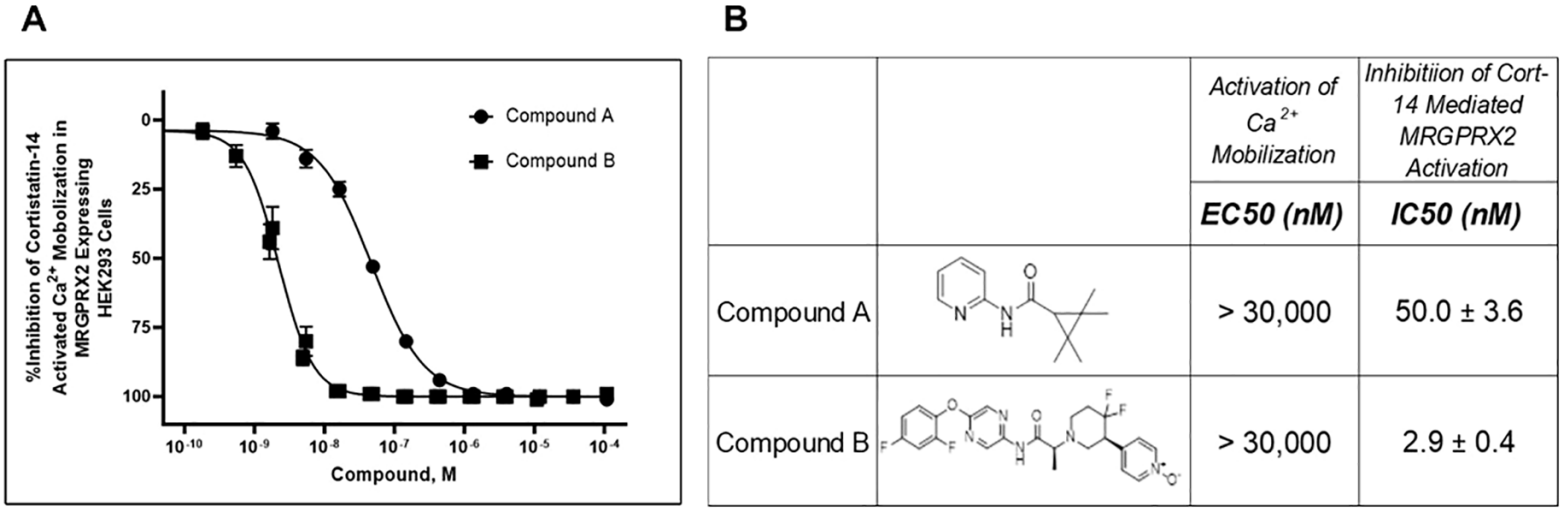
Identification of two structurally distinct MRGPRX2 antagonists: Compound **(A)** and Compound **(B)**. Calcium mobilization assays were performed in FLIPR^TETRA^ (Molecular Devices) with two novel MRGPRX2 antagonists using HEK293 cells overexpressing MRGPRX2 and Gα15 proteins. These compounds had no detectable agonist activity up to the highest concentration tested (EC_50_ > 30,000 nM). Dose-dependent inhibition **(A)** of Cortistatin 14–mediated MRGPRX2 activation was measured, and antagonist potencies **(B)** are presented as mean ± SEM of 66 or 22 independent experiments for Compound A and Compound B, respectively.

**Figure 2:**
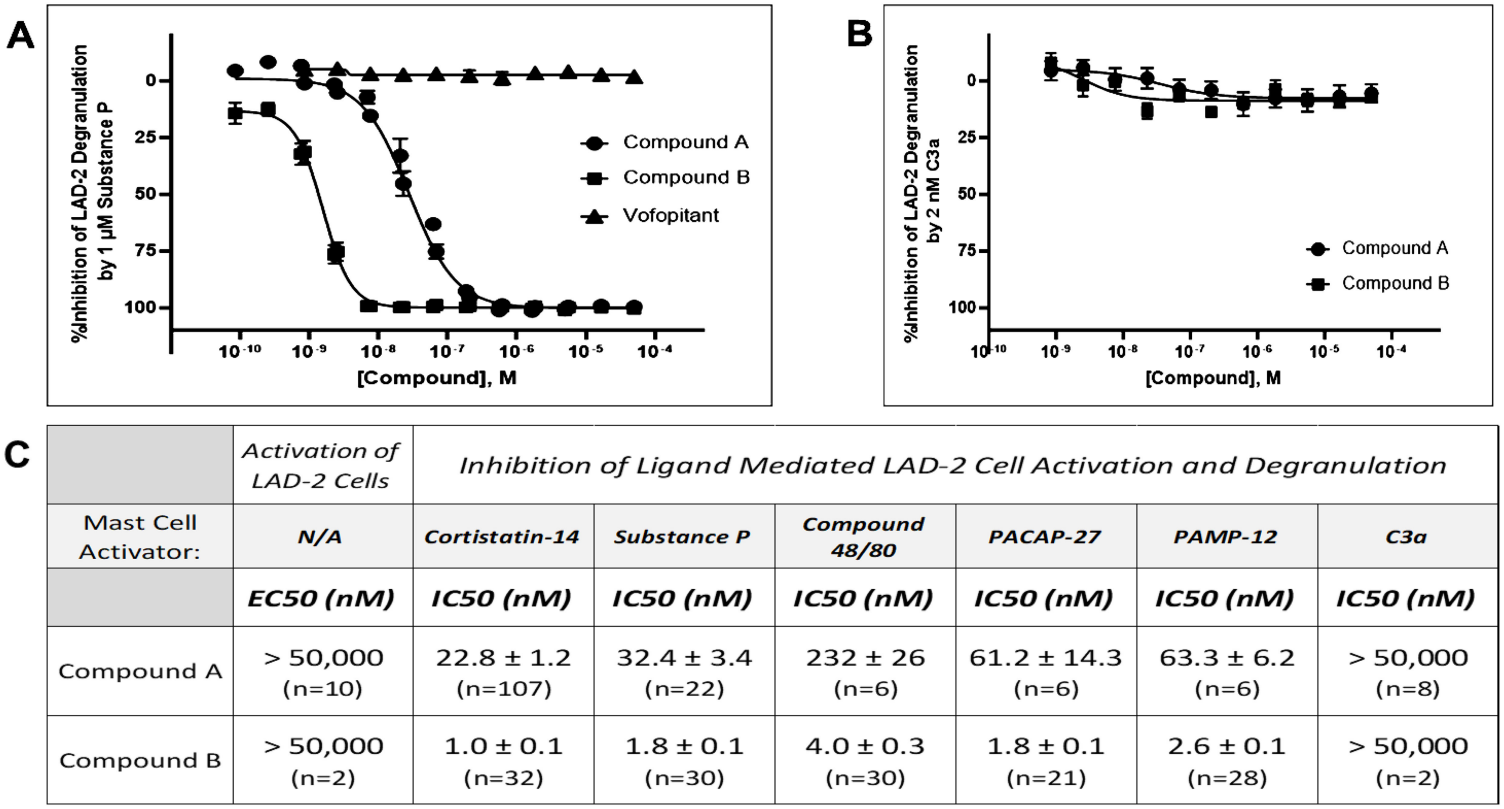
Substance P–stimulated LAD2 degranulation is selectively blocked by MRGPRX2 antagonists but not by the NK1 receptor antagonist vofopitant. **(A)** Unlike Compound A (IC_50_ = 32.4 nM) and Compound B (IC_50_ = 1.8 nM), vofopitant (n = 8), a potent NK1 receptor antagonist, has no influence on Substance P (1 μM, at approximately EC_80_)–mediated mast cell degranulation as measured by β-hexosaminidase release assays. **(B)** Concentration response curves demonstrating lack of an effect of increasing concentrations of either Compound A or Compound B on C3a-stimulated LAD2 human mast cell degranulation. **(C)** Although Compound A or Compound B was inactive as agonist up to the highest concentration tested (EC_50_ > 50,000 nM), they were potent inhibitors on MRGPRX2 ligand-mediated mast cell degranulation. Antagonism on MRGPRX2 is observed across all agonists tested in LAD2 mast cells, demonstrating little or no ligand dependence for either antagonist. Potency data are presented as mean ± SEM. Five MRGPRX2 agonists—Cortistatin 14, Substance P, Compound 48/80, PACAP-27, and PAMP-12—were used in the experiment. Representative dose response curves for these MRGPRX2 agonists are showed in **Supplementary Figure 1**. C3a is an agonist for another mast cell activating receptor, C3aR1. Concentrations at approximately 80% maximum activation (EC_80_) were calculated and used for each mast cell activator in these experiments with Compound A or Compound B.

**Figure 3:**
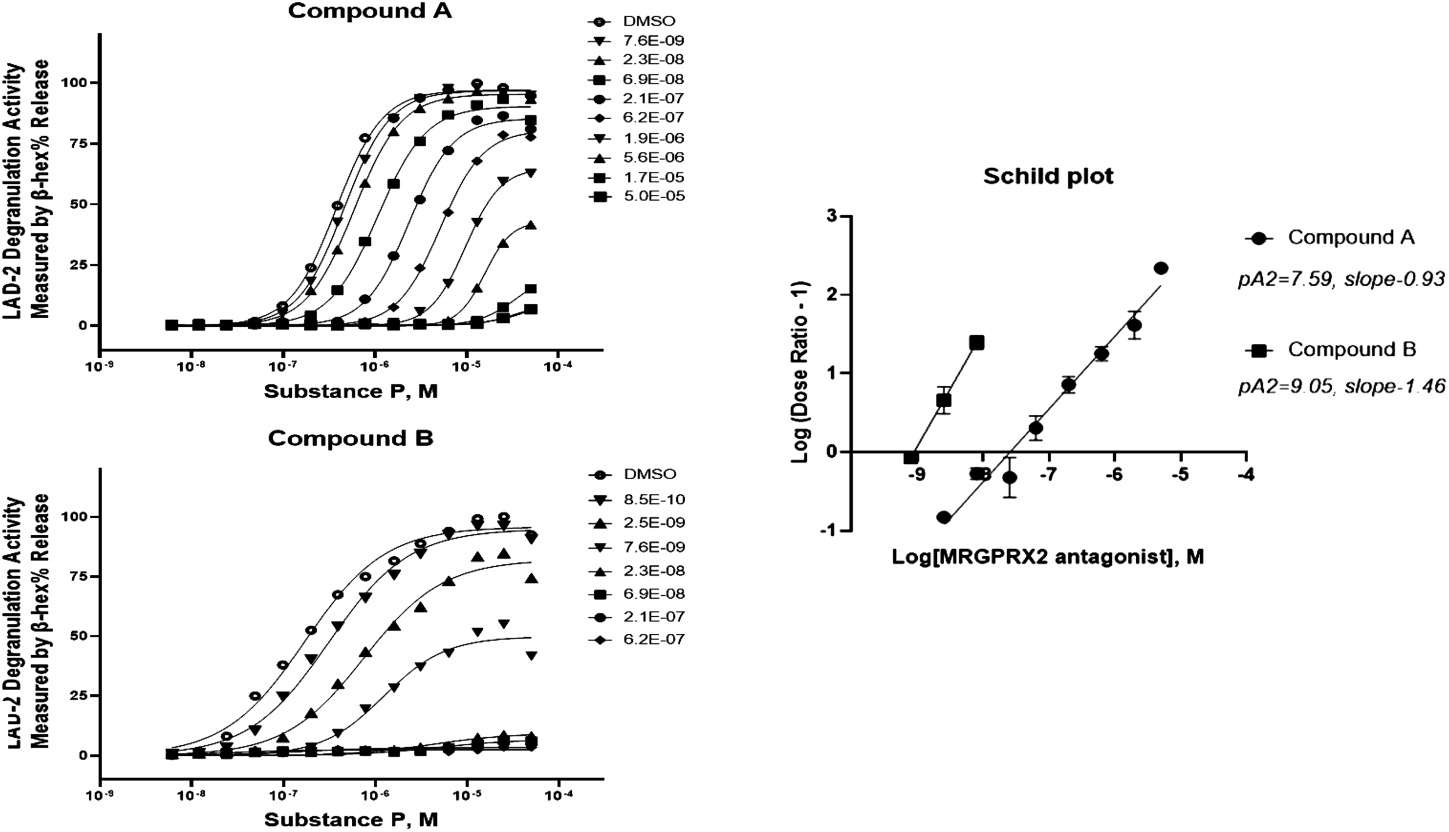
Both Compound A and Compound B exhibit insurmountable inhibition of Substance P–stimulated LAD2 mast cell degranulation. Concentration response curves of Substance P–mediated mast cell degranulation were studied in the absence (DMSO control) or the presence of increasing concentration of antagonists. Schild analysis was performed with the linear regression plot of Log[MRGPRX2 antagonist] at (x)-coordinate and Log (Dose Ratio − 1) at (y)-coordinate. The apparent antagonist affinity determined from the curve shift analyses indicated a mean pA2 value of 7.59 (Schild slope of 0.93, n = 6) or 9.05 (Schild slope of 1.46, n = 7) for Compound A and Compound B, respectively.

**Figure 4:**
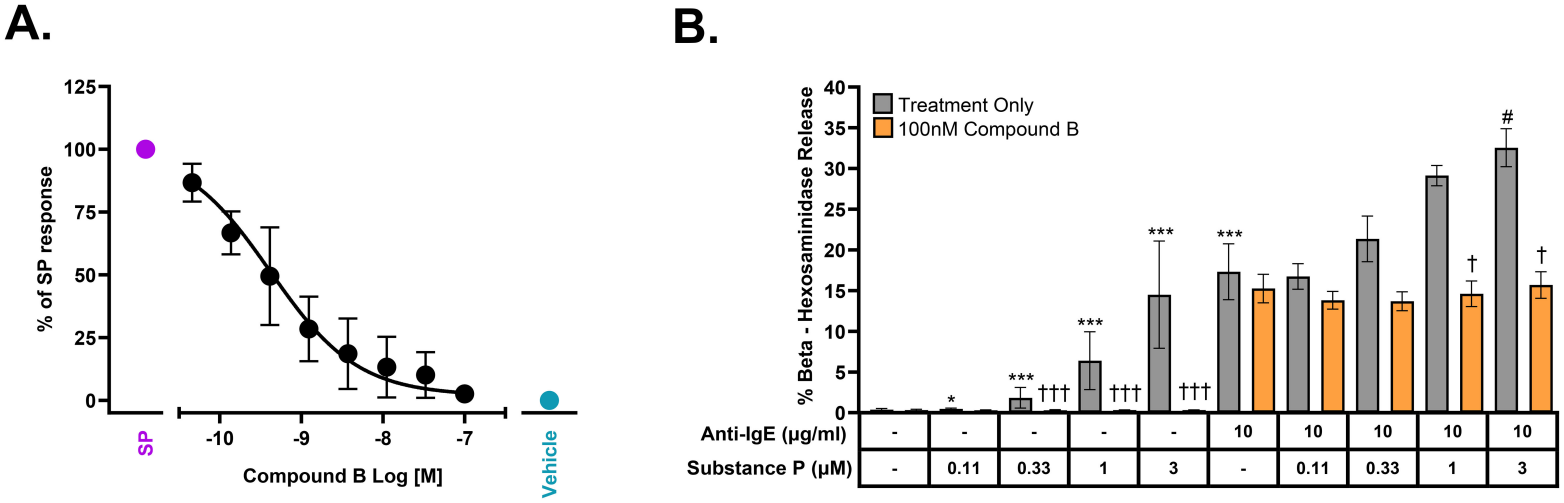
Antagonism of MRGPRX2 by Compound B in freshly isolated human skin mast cells in the presence or absence of FcεR1 activation. **(A)** Concentration-dependent inhibition of Substance P (10 μM, approximately EC_90_)–stimulated mast cell tryptase release by Compound B with a pIC_50_ = 9.38 ± 0.23 (IC_50_ = 0.42 nM, n = 4 donors). **(B)** Compound B selectively inhibits MRGPRX2-dependent augmentation of FcεR1/MRGPRX2 dual-stimulation–mediated β-hexosaminidase release (mean ± SEM, n = 3 donors). Statistical differences determined with two-sided t-tests using the marginal means based on the linear mixed effects model performed on log10-transformed pooled data; single treatments compared to untreated control (*), co-treatments compared to anti-IgE alone (10 μg/mL) (#), or all treatments compared to matched no compound controls (†) are displayed: Substance P treatments, 0.012 µM and 0.037 µM used in statistical analysis, data not shown (FDR-adjusted P-value, */#/† ≤ 0.05, **/##/†† ≤ 0.005, and ***/###/††† ≤ 0.001).

The activity values of MRGPRX2 antagonists in **Figures 1**–**3** are presented as Mean ± SEM (standard error of mean) when applicable. The dataset analyzed for % β-hexosaminidase release in **Figure 4B** (Antagonism of MRGPRX2 by Compound B in freshly isolated human skin mast cells in the presence or absence of FcεR1 activation) consisted of three individual experiments/donors with 28 treatments and two technical replicates per treatment and donor. The percentage raw data were log10-transformed for variance stabilization and modeled with a linear mixed effects model that included a fixed treatment effect (28 levels of treatment, a subset shown) and a random donor effect. Statistical differences between treatment groups were assessed via 33 *post-hoc* two-sided t-tests. An FDR correction with a p-value threshold of 0.05 was applied to guard against false positive findings. The packages lme4 and emmeans in R were used to fit the model and perform the *post-hoc* tests, respectively.

**Figure 5:**
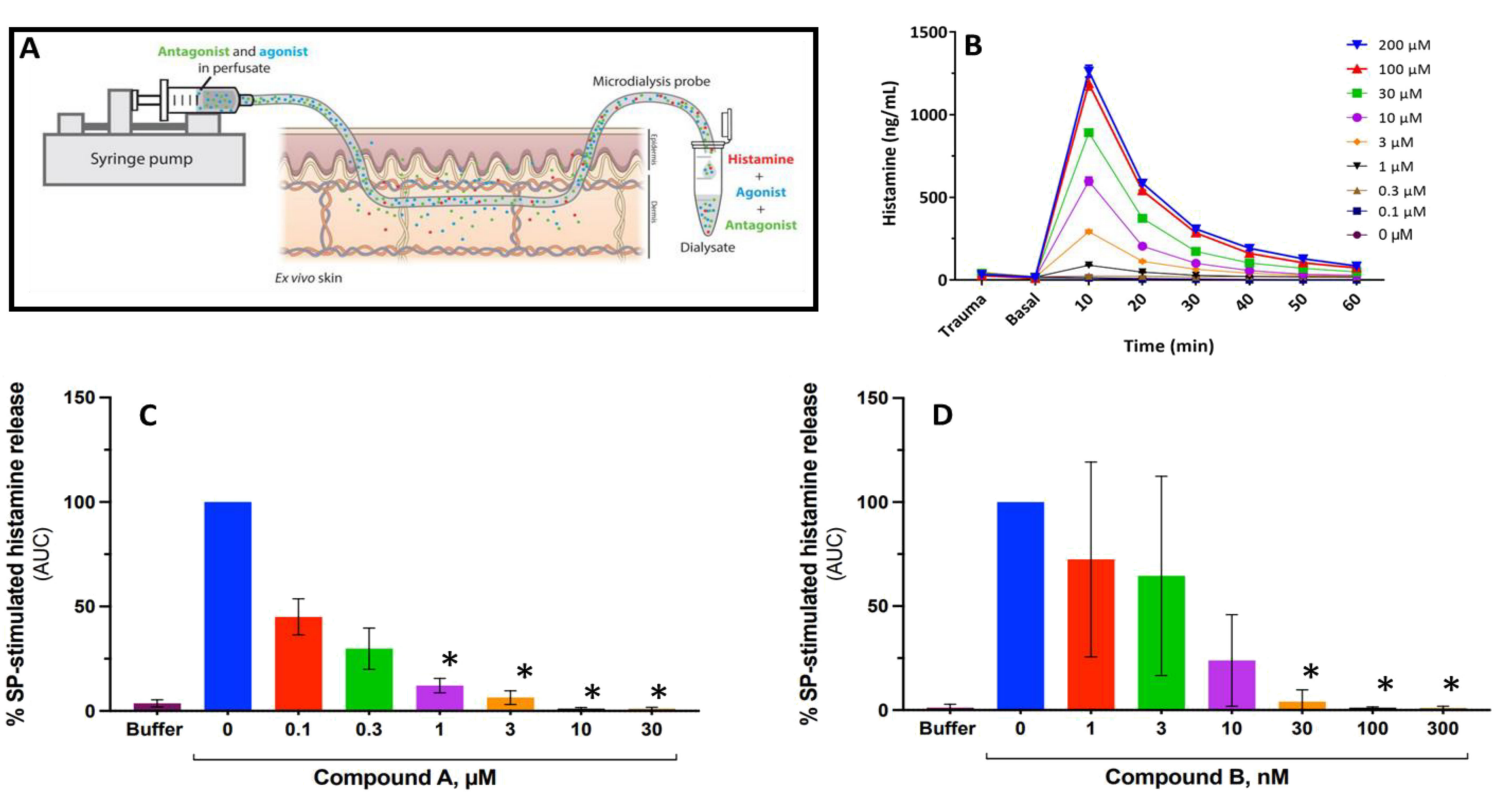
Antagonism of MRGPRX2 blocks Substance P–stimulated histamine release in a perfused human *ex vivo* skin preparation. **(A)** Schematic describing how retrodialysis enables intradermal delivery of the compound of interest via the microdialysis probe while allowing for simultaneous recovery of biomarkers. **(B)** Time course of Substance P–mediated histamine release from a representative experiment with peak 10 min after stimulus. **(C, D)** Substance P–stimulated histamine release expressed as area under the curve (AUC) normalized to Substance P (50 μM) alone reflecting total histamine release in the presence or absence of Compound A **(C)** or Compound B **(D)**. Error bars represent mean ± SD for three different donors. *P < 0.01 for antagonist treatments vs. Substance P alone (Dunnett’s adjusted p-value following pseudo log transformation of AUC data).

Statistical analysis of the % SP-stimulated histamine release AUC (area under the curve) values in a perfused human *ex vivo* skin preparation (**Figures 5C, D**) was performed via a mixed effects model, with concentration as a fixed effect and donor as a random effect. A pseudo-log_10_ transformation was applied to the AUC values to stabilize variances. All concentrations were compared to baseline and comparisons were considered significant if the Dunnett’s multiplicity adjusted p-value was less than 0.05. Statistical analysis was performed via proc mixed in SAS 9.4.

**Figure 6:**
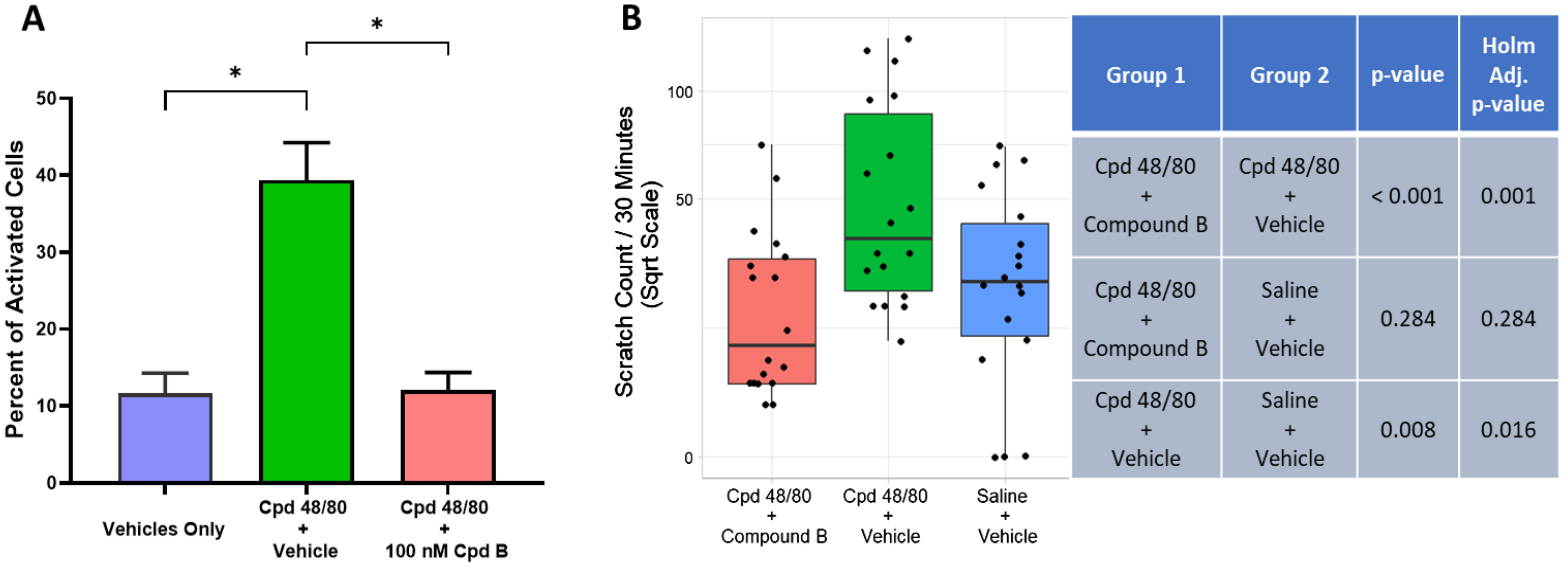
Oral treatment with the potent and selective MRGPRX2 antagonist, Compound B (3 mg/kg), blocks Cpd 48/80–induced itch response using human MRGPRX2 knock-in mice. **(A)** Compound B blocked Cpd 48/80 (1 μg/mL)–mediated activation of peritoneal mast cells isolated from human MRGPRX2 KI mice (n ≥ 4, mean ± SEM), *P < 0.01; Student’s t-test. **(B)** Compound B administration achieved significant blockade of Cpd 48/80–induced itch. Average ± SD blood-free concentration of antagonist measured at end of “itch” observation period was 19 ± 16 nM Compound B.

Statistical analysis for the itch behavioral study of human MRGPRX2 knock-in mice (**Figure 6B**) was conducted using a mixed effects model with treatment group as a fixed effect and date of measurement as a random effect. A square root transformation was applied to count values for variance stabilization. Holm adjusted p-values were reported with significance declared when the adjusted p-value was less than 0.05. The lmer package in R was used to perform statistical analysis.

## Results

### Identification of potent and selective MRGPRX2 antagonists

Direct interaction of antagonists with MRGPRX2 was demonstrated using both HEK293 (human embryonic kidney) cells overexpressing human MRGPRX2 as well as human mast cells expressing endogenous MRGPRX2. Two different *in vitro* human mast cell systems were studied: (a) a human mast cell line, LAD2, established from a patient with mast cell sarcoma; and (b) mast cells freshly isolated from human skin. MRGPRX2 antagonist IC_50_ values were determined in the LAD2 mast cell degranulation assay using approximately EC_80_ concentrations for five different MRGPRX2 activating ligands (see **Supplementary Figure 1**).

Two-potent, heteroaryl acetamide, antagonists with no detectable agonist activity (up to 30 μM tested) were initially characterized in the HEK293 Cortistatin 14–mediated calcium mobilization assay (**Figure 1**). Compound A was an original hit identified in the high-throughput screening effort with Ca^2+^ mobilization assay using the HEK293-MRGPRX2/Gα15 cells. It is a small and ligand efficient antagonist molecule. However, the pharmacokinetic (PK) profile of Compound A limits its utility to *in vitro* and *ex vivo* assays only. Compound B is the result of multiple iterations of chemical modification on an original High Throughput Screening (HTS) hit and had good *in vivo* PK profile and improved potency, making it an excellent tool for all study conditions including studies in animal models. In this assay, Compound A inhibited the cortistatin-mediated response in a concentration-dependent manner with an IC_50_ of 50 nM, whereas Compound B similarly inhibited the cortistatin-mediated response with an IC_50_ of 2.9 nM (**Figure 1**).

Compound A and Compound B were then further characterized in a mast cell degranulation assay with LAD2 cells. Neither has detectable activation activity (up to 50 μM tested). Both compounds demonstrating concentration-dependent inhibition of Cortistatin 14–mediated degranulation with IC_50_’s of 22.8 nM and 1.0 nM, respectively (**Figure 2C**). When another known MRGPRX2 agonist, SP, was used to activate LAD2 cells, both compounds demonstrating a concentration-dependent inhibition with IC_50_’s of 32.4 nM and 1.8 nM, respectively (**Figures 2A, C**). While complete blockade was achieved with these two MRGPRX2 antagonists, SP-mediated degranulation was unaltered by a potent neurokinin 1 (NK1) receptor antagonist, vofopitant (16), demonstrating that all of SP’s actions on mast cell is mediated through activation of MRGPRX2. Moreover, while potently blocking the mast cell degranulation response to SP, Compound A or B had no influence on the degranulation response to complement C3a, which mediates its effects through its own C3aR1 receptor on mast cells (**Figure 2B**). In addition to achieving excellent selectivity over another mast cell GPCR, these data also demonstrate that Compound A and Compound B does not interfere with cellular processes controlling degranulation. In addition, antagonism by Compound A and Compound B was not influenced by the MRGPRX2 agonists tested, i.e., there was little ligand-dependent effort when evaluated against five different MRGPRX2 activating ligands (**Figure 2C**).

When tested against SP in LAD2 degranulation assays, antagonism by Compound A was shown to be partly surmountable in nature in that it demonstrated parallel rightward shift of agonist concentration response curves with moderate alteration of the maximal response (**Figure 3**). In contrast, Compound B is clearly an insurmountable MRGPRX2 antagonist. For example, 20 nM Compound B was found to abolish the degranulation response to the highest concentration of SP studied, 50 μM. As anticipated, the Schild Plot slope for Compound B was steeper than that for Compound A with a corresponding pA2 of 9.05 (**Figure 3**). This non-competitive mechanism has important implications for predicting the efficacious dose for clinical evaluation because, for example, even at a low concentration of 7.6 nM, Compound B was noted to dampen SP’s maximal response.

Both Compound A and Compound B demonstrated excellent selectivity in off-target screening for *in vitro* secondary pharmacology that included GPCRs, ion channels, and enzymes as targets. When tested against MRGPRX1, the GPCR with closest sequence homology with MRGPRX2, both Compound A and Compound B showed no detectable agonist or antagonist activity (**Supplementary Table 2**). Compound A and Compound B also demonstrated excellent selectivity against 19 other GPCRs as listed in **Supplementary Table 2**. Among them, the NK1 receptor with which SP is the canonical and high-affinity endogenous ligand. Both Compound A and Compound B were also inactive in other *in vitro* assays on a number of ion channel and enzyme targets that were part of the overall human safety prediction panel (data not shown).

### Substance P activates freshly isolated human skin mast cells, and its activity is additive with FcεR1-mediated degranulation

Although the human mast cell line LAD2 expresses functional MRGPRX2, these cells represent immature mast cells and do not, for example, express tryptase. Thus, it was important to show similar efficacy in mature mast cells freshly isolated from human skin which express both MRGPRX2 and tryptase. **Figure 4A** demonstrates that Compound B is a potent antagonist of SP-mediated tryptase release from mature human mast cells with a pIC_50_ of 9.38 (0.42 nM). We also sought to determine whether concomitant activation of both MRGPRX2 and the IgE receptor, FcεR1, would be additive with respect to tryptase release. **Figure 4B** clearly demonstrates that there is, at the very least, an additive mast cell degranulation response when both receptors are activated in unison with addition of Compound B specifically blocking the SP response but not the response to anti-IgE.

Because basophils have been reported to express active MRGPRX2 (13), we wanted to demonstrate similar blockade of an SP-mediated response in this disease-relevant immunocyte. However, no SP response could be detected in freshly prepared human basophils (**Supplementary Figure 2**). Although anti-IgE could stimulate a robust histamine release in a concentration-dependent manner, no SP response could be elicited by the concentrations tested (0.2–25 μM). This was the case whether basophils were isolated from either non-allergic or allergic individuals, with the latter demonstrating an augmented response to anti-IgE (**Supplementary Figure 2**).

### MRGPRX2 is solely responsible for SP-stimulated histamine release from perfused human skin

The antagonist effect of MRGPRX2 antagonists on SP-induced histamine release was assessed in a human *ex vivo* skin model using abdominal tissue obtained from three different individual donors (**Figure 5**). Intradermal delivery of SP by retrodialysis resulted in a concentration-dependent release of histamine that peaked within the first 10 min (**Figure 5B**). On average, a maximal response to SP was demonstrated at concentrations ≥ 100 μM.

Perfusion with either antagonist alone did not induce a release of histamine from skin-resident mast cells. Pre-treatment with both antagonists demonstrated a concentration-dependent inhibition of histamine release with complete blockade of the SP (50 μM) response achieved at 3 μM or 30 nM for Compound A and Compound B, respectively (**Figures 5C, D**).

### MRGPRX2 antagonism attenuates itch

The MRGPRX2 antagonists used in the studies here were not active against any animal ortholog of human MRGPRX2 tested (**Supplementary Table 1**). Due to the human selectivity profile of these MRGPRX2 antagonists, it was necessary to use humanized mice to assess PK/pharmacodynamic relationships. Compound B was used in this *in vivo* study because, unlike Compound A, it demonstrated good oral bioavailability. Compound B (100 nM) was shown to completely antagonize Cpd 48/80–mediated activation of peritoneal mast cells isolated from human MRGPRX2 knock-in (KI) mice (**Figure 6A**), consistent with mast cell expression of functional human MRGPRX2 as previously demonstrated (15). These mice were used to provide initial proof of target engagement of MRGPRX2 antagonists in an established behavioral scratching model induced by intradermal injection of a MRGPRX2 agonist, Cpd 48/80 (17). **Figure 6B** demonstrates that oral gavage of Compound B (3 mg/kg) was able to completely block the itch response to Cpd 48/80 in human MRGPRX2 KI mice, whereas no effect was observed in similar behavioral study using wild-type mice (**Supplementary Figure 3**).

## Discussion

Prior studies have demonstrated that the MRGPRX2 receptor is expressed solely by connective tissue mast cells (12). This is consistent with the current study that also included an investigation of basophils. Unlike LAD2 mast cells or freshly isolated human skin mast cells, SP could not elicit a functional response by blood basophils even following anti-IgE pre-incubation. This lack of a response to SP was observed in basophils isolated from both non-allergic and allergic individuals. Although these findings agree with other studies showing the lack of a functional MRGPRX2 receptor in resting basophils isolated from blood, unlike others (18), we were unable to demonstrate that a functional basophil MRGPRX2 receptor can be upregulated after prior stimulation with anti-IgE.

The knowledge that neuropeptides represent the most potent agonists for MRGPRX2 is biologically significant, because it has been known for decades that mast cells uniquely make close connections with sensory nerves and that these mast cells are increased in disease tissues (19). Thus, within these neuroimmune synapses, MRGPRX2-expressing mast cells are anatomically primed to respond to neuropeptides such as SP released by nearby sensory neurons. It has been proposed that different sensory nerves might interact with mast cells in specific ways. For example, mast cell interaction with peptidergic nerves in skin results in inflammation, pruritis, and pain (i.e., neurogenic inflammation), whereas interaction with non-peptidergic nerves has a regulatory role (20). In support of the former, it has been postulated that SP released from sensory nerves may play a key function in a positive feedback loop in sensory neuron transmission by stimulating adjacent mast cells to release tryptase. As a result, tryptase-mediated cleavage and activation of the protease-activated receptor 2 (PAR2) augment further neuropeptide release via sensitization of TRPV1-expressing neurons (21). Consistent with this proposal has been the demonstration that TRPV1^+^/SP^+^ nociceptor–MRGPRB2 mast cell clusters are critical in driving the clinical score of a severe preclinical model of atopic dermatitis (9). In this model of atopic dermatitis, cysteine protease activity within house dust mite extracts was necessary for sensitizing TRPV1^+^ neurons. Moreover, tryptase released by MRGPRX2/MRGPRB2 activation is also involved in the release of type 2 cytokines, which contributes to the inflammation associated with atopic dermatitis (22).

A study investigating the cutaneous gene expression profile of primates with nonhistaminergic itch demonstrated that the aforementioned MRGPRX2 “pathway” (i.e., SP, MRGPRX2, tryptase, PAR2, and TRPV1) was upregulated (23). These differentially expressed genes mirrored the “itchscriptome” observed in human chronic pruritus (24). Also, of note, the number of MRGPRX2-expressing mast cells is increased in the lesional skin of patients with, for example, chronic spontaneous urticaria and prurigo nodularis, two skin disorders characterized by intense itch (24–26). This prompts the question as to where these MRGPRX2-expressing mast cells originated, proliferation of resident connective tissue mast cells, or recruitment of circulating mast cell progenitors. The latter cannot be ruled out because immature progenitor mast cells express MRGPRX2, whether it be LAD2 cells or freshly isolated circulating human progenitors [(West et al.)^1^ *manuscript accepted for publication in iScience*)]. One possible mechanism would be chemotaxis of circulating, MRGPRX2-expressing, progenitor mast cells down a neuropeptide concentration gradient (e.g., SP) generated by activated skin resident sensory neurons. The latter possibility is consistent with the observation that circulating progenitors, normally excluded from steady-state skin, are recruited only into inflamed skin where they then clonally expand alongside skin resident mast cells (27).

Here, we describe the properties of two new potent and selective MRGPRX2 antagonists, Compound A and Compound B, with the latter demonstrating excellent sub-nM potency in freshly isolated human skin mast cells. These antagonists were also shown to completely block SP-mediated histamine release in a concentration-dependent manner using a unique *ex vivo* human skin preparation that incorporated microdialysis. This study provides pharmacological evidence that MRGPRX2 is solely responsible for mediating an SP response in a human tissue, in this instance, skin. Finally, human MRGPRX2 knock-in mice were used to provide initial proof of target engagement of MRGPRX2 antagonists in the skin using an established behavioral scratching model. Of note, a blood sample taken at the very end of the pre-determined scratching period demonstrated that the observed average blood free level of Compound B was 19 nM (data not shown). This is important and consistent with *in vitro* and *ex vivo* data presented in this manuscript supporting that a blood free concentration of ≥ 20 nM Compound B would be sufficient to completely block a response driven by MRGPRX2 activation. Thus, taken together, these data support the future investigation of MRGPRX2 receptor antagonists in skin disorders.

## Data Availability

The original contributions presented in the study are included in the article/**Supplementary Material**. Further inquiries can be directed to the corresponding author.

## Footnotes

1 West PW, Cheret J, Bahri R, Kiss O, Wu Z, Macphee CH, et al. (In press). The MRGPRX2-Substance P pathway regulates mast cell migration. iScience. doi: 10.1016/j.isci.2024.110984
