# Supplementary material for: Pharmacological blockade of the mast cell MRGPRX2 receptor supports investigation of its relevance in skin disorders
Source: Front Immunol. 2024 Oct 18;15:1433982. doi: 10.3389/fimmu.2024.1433982 (PMC11527646; doi:10.3389/fimmu.2024.1433982)
Supplement: Supplementary file 1 [file Image1.pdf]

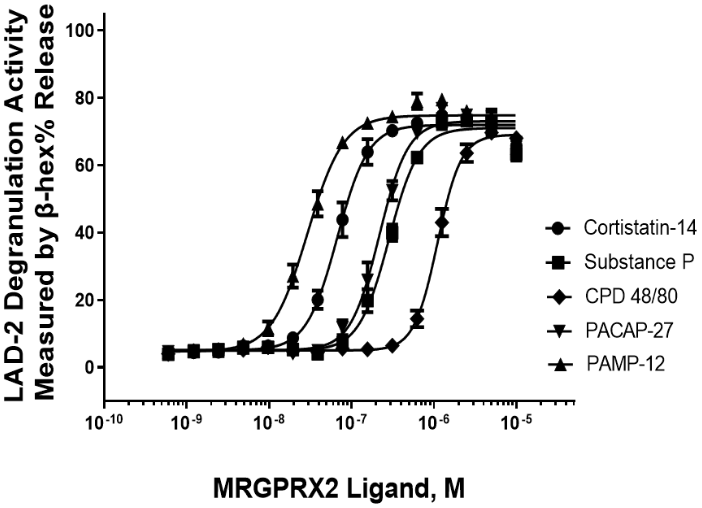

Supplemental Figure 1: LAD2 mast cell degranulation concentration response curves to various MRGPRX2 agonists. Data are showed as representative dose response curves for Cortistatin 14 (EC_{50} = 67 nM), Substance P (EC_{50} = 285 nM), Cpd 48/80 (EC_{50} = 1,108 nM), PACAP-27 (EC_{50} = 218 nM) and PAMP-12 (EC_{50} = 29 nM).
