# Supplementary material for: Pharmacological blockade of the mast cell MRGPRX2 receptor supports investigation of its relevance in skin disorders
Source: Front Immunol. 2024 Oct 18;15:1433982. doi: 10.3389/fimmu.2024.1433982 (PMC11527646; doi:10.3389/fimmu.2024.1433982)
Supplement: Supplementary file 2 [file Image2.pdf]

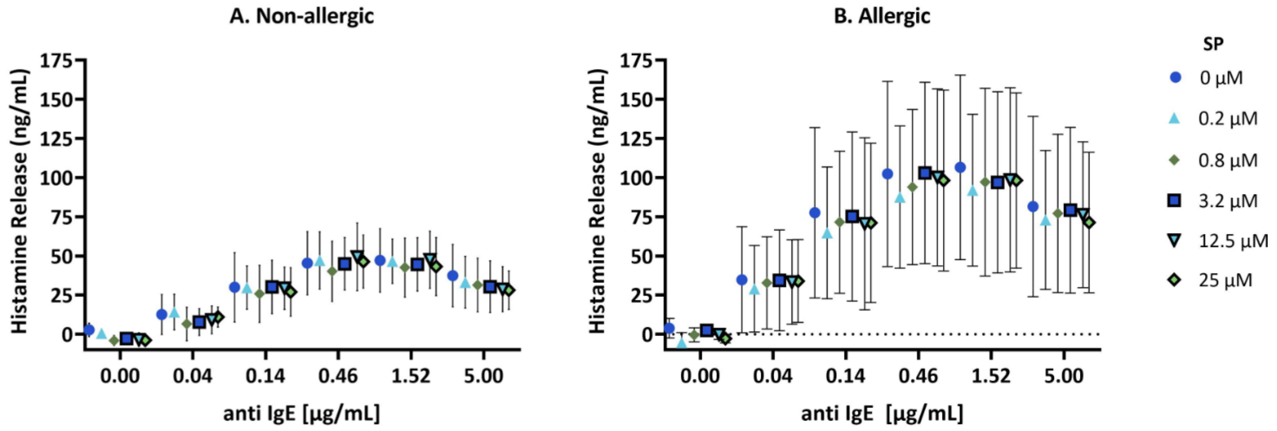

Supplemental Figure 2: Human blood basophils do not respond to Substance P with or without Anti-IgE pre-stimulation. Histamine release measured in blood samples pre-incubated with various concentrations of anti-lgE for 30 mins, followed by 1 hour incubation with various concentrations of Substance P. The data represents the mean ± SD from either five non-allergic (A) or five allergic (B) donors challenged with the corresponding concentrations of anti-IgE and Substance P.
