# Supplementary material for: Pharmacological blockade of the mast cell MRGPRX2 receptor supports investigation of its relevance in skin disorders
Source: Front Immunol. 2024 Oct 18;15:1433982. doi: 10.3389/fimmu.2024.1433982 (PMC11527646; doi:10.3389/fimmu.2024.1433982)
Supplement: Supplementary file 3 [file Image3.pdf]

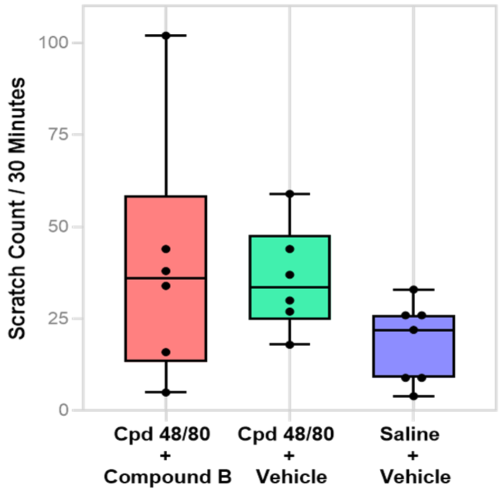

Group 1	Group 2	p-value
Cpd 48/80 + Compound B	Cpd 48/80 + Vehicle	0.795
Cpd 48/80 + Compound B	Saline + Vehicle	0.140
Cpd 48/80 + Vehicle	Saline + Vehicle	0.031*

Supplemental Figure 3: Oral treatment with the potent and selective human MRGPRX2 antagonist, Compound B (3 mg/kg), did not block Cpd 48/80 – induced itch response in wild-type mice. The itch behavioral study for wild-type mice (C57BL6J strain) was performed the same way as descripted in the Methods section as for human MRGPRX2 knock-in mice. Compound 48/80 treatment (35.8 +/- 5.4, n=6) significantly increased the scratching counts compared to saline treated control (18.4 +/- 3.8, n=7) mice (mean +/- SEM; *P < 0.05, Student's t test). As expected, no effect on the scratching behavior (39.8 +/- 12.6, n=6; P=0.795) was observed by oral administration of Compound B to the Compound 48/80 treated mice.
