# Supplementary material for: Pharmacological blockade of the mast cell MRGPRX2 receptor supports investigation of its relevance in skin disorders
Source: Front Immunol. 2024 Oct 18;15:1433982. doi: 10.3389/fimmu.2024.1433982 (PMC11527646; doi:10.3389/fimmu.2024.1433982)
Supplement: Supplementary file 4 [file Table1.pdf]

	Agonist and Antagonist Activities of Compounds in FLIPR Assays with U2OS Cells Expressing Human MRGPRX2 or its Animal Orthologs														
Species	*Human*		*Monkey: Cynomolgus macaques*		*Monkey: Rhesus macaques*		*Dog (Beagle): Canis lupus*		*Dog (Boxer): Canis familiaris*		*Mouse (MrgB2): Mus musculus*		*Rat (MrgB3): Rattus norvegicus domestica*	
	Agonist ***EC50, µM***	*Antagonist* ***IC50, nM***	*Agonist* ***EC50, µM***	*Antagonist* ***IC50, µM***	*Agonist* ***EC50, µM***	*Antagonist* ***IC50, µM***	*Agonist* ***EC50, µM***	*Antagonist* ***IC50, µM***	*Agonist* ***EC50, µM***	*Antagonist* ***IC50, µM***	*Agonist* ***EC50, µM***	*Antagonist* ***IC50, µM***	*Agonist* ***EC50, µM***	*Antagonist* ***IC50, µM***
Compound A	> 100 (n=7)	44.6 ± 10.5 (n=7)	1.4 (n=1)	17.8 ± 0.4 (n=2)	0.89 ± 0.13 (n=2)	42.6 ± 13.1 (n=3)	> 100 (n=3)	> 80 (n=3)	> 100 (n=3)	> 100 (n=2)	> 100 (n=2)	> 100 (n=2)	> 100 (n=2)	> 40 (n=1)
Compound B	> 100 (n=31)	3.6 ± 0.2 (n=13)	11.5 ± 1.3 (n=8)	38.2 ± 1.9 (n=11)	6.4 ± 0.5 (n=10)	14.0 ± 0.6 (n=11)	> 80 (n=9)	> 100 (n=11)	> 100 (n=11)	> 80 (n=8)	> 100 (n=10)	> 100 (n=10)	> 100 (n=10)	> 100 (n=10)

Supplemental Table 1: Compound A and Compound B are human MRGPRX2 selective antagonists. Antagonist and agonist activities of the two compounds were determined in calcium mobilization assays for human MRGPRX2 and its animal orthologs from various species. The activities were measured in FLIPR^{TETRA} (Molecular Devices) instrument using U2OS cells transduced with BacMam virus for transient expression of the relevant receptors. Data are presented as mean ± SEM when applicable. No agonist activity was detected on human MRGPRX2 for both Compound A and Compound B up to the highest concentration (100 µM) tested. In the antagonist assay against Cortistatin 14 (at approx. EC_{80}), IC_{50}'s of 44.6 nM and 3.6 nM, were detected for Compound A and B, respectively. These results are consistent with those obtained in calcium mobilization assays using a different cell line, i.e. HEK293 cells with stable expression of MRGPRX2 and Gα15 proteins (Fig. 1). On monkey MRGPRX2 receptors (both *Cynomolgus macaques* and *Rhesus macaques*), weak agonist activities were detected for both Compound A and Compound B. Though very weak antagonist activities were also reported, they were likely due to receptor desensitization upon activation and not real antagonism. No agonist nor antagonist activity was detected by Compound A or Compound B in assays with other orthologs of human MRGPRX2, namely the dog MRGPRX2 (beagle & boxer), mouse MrgB2 and rat MrgB3. Taking together, Compound A and Compound B demonstrated human MRGPRX2 specific antagonist activity.
