# Supplementary material for: Pharmacological blockade of the mast cell MRGPRX2 receptor supports investigation of its relevance in skin disorders
Source: Front Immunol. 2024 Oct 18;15:1433982. doi: 10.3389/fimmu.2024.1433982 (PMC11527646; doi:10.3389/fimmu.2024.1433982)
Supplement: Supplementary file 5 [file Table2.pdf]

frontiers

GPCR Target Name	Gene symbol	Mode of Activity	*Agonist Activity (* ***EC50*** *) or Antagonist Activity (* ***IC50*** *), nM*	
**GPCR Target Name**	**Gene symbol**	**Mode of Activity**	**Compound-A**	**Compound-B**
A_{2A} receptor	ADORA2A	Agonism	> 100,000	> 100,000
α_{1B}-Adrenoreceptor	ADRA1B	Antagonism	> 25,000	> 25,000
α_{2C}-Adrenoreceptor	ADRA2C	Agonism	n/a	> 100,000
ß$_2$-Adrenoreceptor	ADRB2	Agonism	n/a	> 100,000
ß$_2$-Adrenoreceptor	ADRB2	Antagonism	n/a	> 100,000
CB_1 receptor	CNR1	Agonism	> 25,000	> 25,000
D_1 receptor	DRD1	Antagonism	> 100,000	> 100,000
D_2 receptor	DRD2	Agonism	n/a	> 100,000
D_2 receptor	DRD2	Antagonism	n/a	> 100,000
H_1 receptor	HRH1	Antagonism	> 25,000	> 25,000
H_3 receptor	HRH3	Antagonism	n/a	> 50,000
M_1 receptor	CHRM1	Agonism	> 50,000	> 50,000
M_1 receptor	CHRM1	Antagonism	> 50,000	> 50,000
M_2 receptor	CHRM2	Agonism	> 50,000	> 50,000
M_2 receptor	CHRM2	Antagonism	> 50,000	> 50,000
MRGPRX1	MRGPRX1	Agonism	> 100,000	> 100,000
**MRGPRX1**	**MRGPRX1**	**Antagonism**	**> 100,000**	**> 100,000**
**NK_1 receptor**	**TACR1**	**Antagonism**	**> 25,000**	**> 25,000**
κ-Receptor	OPRK1	Agonism	> 100,000	> 100,000
μ-Receptor	OPRM1	Agonism	n/a	> 100,000
V_{1A} receptor	AVPR1A	Antagonism	> 50,000	> 50,000
5-HT_{1B} receptor	HTR1B	Agonism	11,900	> 50,000
5-HT_{2A} receptor	HTR2A	Agonism	> 100,000	> 100,000
5-HT_{2A} receptor	HTR2A	Antagonism	41,400	> 100,000
5-HT_{2B} receptor	HTR2B	Agonism	27,100	> 100,000
5-HT_{2C} receptor	HTR2C	Agonism	> 100,000	> 100,000
5-HT_{2C} receptor	HTR2C	Antagonism	> 100,000	> 50,000
MRGPRX2	MRGPRX2	Agonism	> 30,000	> 30,000
**MRGPRX2**	**MRGPRX2**	**Antagonism**	**50**	**2.9**

Supplemental Table 2: Compound A and Compound B are highly selective MRGPRX2 antagonists, with little activity detected on other GPCRs. Antagonist and/or agonist activities of the two compounds were determined in cellular assays for 20 human G-protein coupled receptors (GPCR) beside MRGPRX2. Recombinant over-expression systems in mammalian cells were used for all assays listed in this table. If the compound is inactive in the assay, EC_{50} or IC_{50} results were reported as '>' the highest compound concentration allowed in that assay.
